# Spatiotemporal imaging and manipulation of surface plasmons

**DOI:** 10.1515/nanoph-2023-0733

**Published:** 2024-01-11

**Authors:** Kevin T. Crampton, Alan G. Joly, Yu Gong, Patrick El-Khoury

**Affiliations:** Pacific Northwest National Laboratory, Biological Sciences Division, Richland, WA 99354, USA; Pacific Northwest National Laboratory, Physical Sciences Division, Richland, WA 99354, USA; Department of Physics and Astronomy, College of Charleston, 58 Coming Street, Charleston, SC 29424, USA

**Keywords:** nano-optics, photoemission electron micros-copy, photonics, surface plasmon polariton

## Abstract

Surface plasmon polaritons (SPPs) are travelling surface waves that have shown promise for applications in nanophotonics as they provide a direct route toward photon-mediated electrical signal generation – a central paradigm for speeding up and scaling down photonic elements. SPP waves have also generated fundamental interest due to their high-field strength and sub-wavelength confinement, properties that have enabled the surface-enhanced Raman effect. Over the last decade, photoemission electron microscopy (PEEM) has emerged as a pioneering technique for imaging surface electric fields through ultrashort laser pulse mediated electron emission and has therefore become an indispensable tool for characterizing plasmonic phenomena at interfaces in a variety of materials. PEEM offers nanometer spatial resolution and femtosecond temporal resolution, allowing SPPs to be prepared, monitored, and manipulated on the nanometer-femtosecond scale. Through a brief review of recent reports, we aim to introduce PEEM-based SPP imaging and manipulation modalities and highlight their utility in the context of emerging nanoscale and quantum materials science advancements.

## 1 Introduction

Light may be confined as localized electromagnetic fields to nanometer [1]–[5] or even atomic length scales [6]–[8] through the excitation of coherent quasi-particles called surface plasmons. Plasmons are optical excitations sustained through the collective motion of free-charge in materials like noble metals where the interaction between valence electrons and ionic cores may be neglected. This assumption is quite valid for coinage metals (Au, Ag, and Cu) in the visible and near-infrared frequency range where plasmonic phenomena abound and are typically explored. Distinct classes of surface plasmons exist that may be distinguished based on whether they are propagating or localized. The electromagnetic fields associated with the latter case of localized surface plasmons (LSPs) are given by discretized modes governed by the geometry of the subwavelength particles or features that sustain them; they are standing waves. On the other hand, propagating surface plasmon polaritons (SPPs) travel along the interface between metals and dielectrics for some distance until they are damped through metal absorption, scattering, or other channels. Mechanisms of plasmonic decay are discussed in detail in ref. [9]. While LSPs can be prepared through direct illumination, SPPs cannot be excited by free-space radiation because they have more momentum than a photon of the same frequency. A multitude of ways to overcome this mismatch to couple light to SPPs have been devised, for example, through the addition of grating momentum or by scattering at asperities in otherwise planar metal surfaces [10].

The renewed interest in surface plasmon phenomena observed over the past two decades recognizes the utility of SPPs and LSPs for applications in nanophotonics. Extreme subwavelength confinement, near-light speed, and high field strength are among the properties that make plasmons promising as information carriers in the development nano-analogues of photonic circuits. Moreover, the coupling between light and plasmons generally involves linear, coherent interactions which preserve polarization and phase information. This allows SPPs to carry and encode information from light fields as captured by their common portrayal as “light on a wire” [11]–[13]. Other applications such as sensing [14], and surface-enhanced [15]–[18] and tip-enhanced [1], [2], [6], [19] Raman scattering similarly take advantage of the unique properties associated with the plasmonic near-field.

Recent progress in surface plasmon science has been largely enabled through parallel advancements in near-field optical and electron microscopies. Tip-enhanced approaches, for example, rely on surface plasmon localization at the apexes of sharp metal tips. The near-field interaction between tips and metal substrates forms tightly confined cavity plasmons that may be used to sense molecular vibrations at the level of single molecules [6]–[8] or image the local fields sustained by microscopic plasmonic structures [5], [20]–[24]. Scanning near-field optical microscopy (SNOM) has also shown promise in this context. Metal coated tips with or without apertures may broadcast information about proximal plasmonic fields into the far field [25]–[28]. Such processes are mediated by enhanced scattering (LSP) activity and do not strictly require junction plasmons. As an alternate and perhaps more versatile approach, surface electric fields may be imaged directly via ultrashort laser pulse-initiated photoelectron emission using photoemission electron microscopy (PEEM) [29]–[32]. PEEM naturally provides the requisite spatial (nanometer) and temporal (femtosecond) resolution to drive and monitor ultrafast surface dynamics and has been employed extensively in the study of plasmonic phenomena in the near-field [33]–[41].

Here, a brief exposition of recent reports that describe commonly implemented and state-of-the-art PEEM modalities will be used to provide an outlook for broadening our ability to initiate and characterize plasmonic phenomena in metals and materials at the nanoscale. We will focus on time-domain two- and three- pulse interferometric PEEM techniques that have enhanced the repertoire of plasmonic imaging and sensing tools with emphasis on SPP coupling, propagation, and directional manipulation.

## 2 Perspectives and discussion

A basic way to visualize SPP propagation through PEEM involves using a single laser beam for plasmon excitation and interrogation. In such an experiment, the laser impinges on a metal surface at an angle. Coupling to SPPs may be facilitated by imperfections in the metal surface which provide the small amount of momentum required to bridge the SPP-light dispersion gap for forward-propagation. More generally, SPP phase-matching is determined by the photon vacuum wavelength, real part of the complex metal dielectric function, and incidence angle. The incidence angle tunes the photon momentum through its vector projection in the plane of the metal surface. Note, PEEM can also be implemented in normal incidence mode [32]. In this case, the in-plane photon momentum is the same as it is in vacuum.

**Figure 1:**
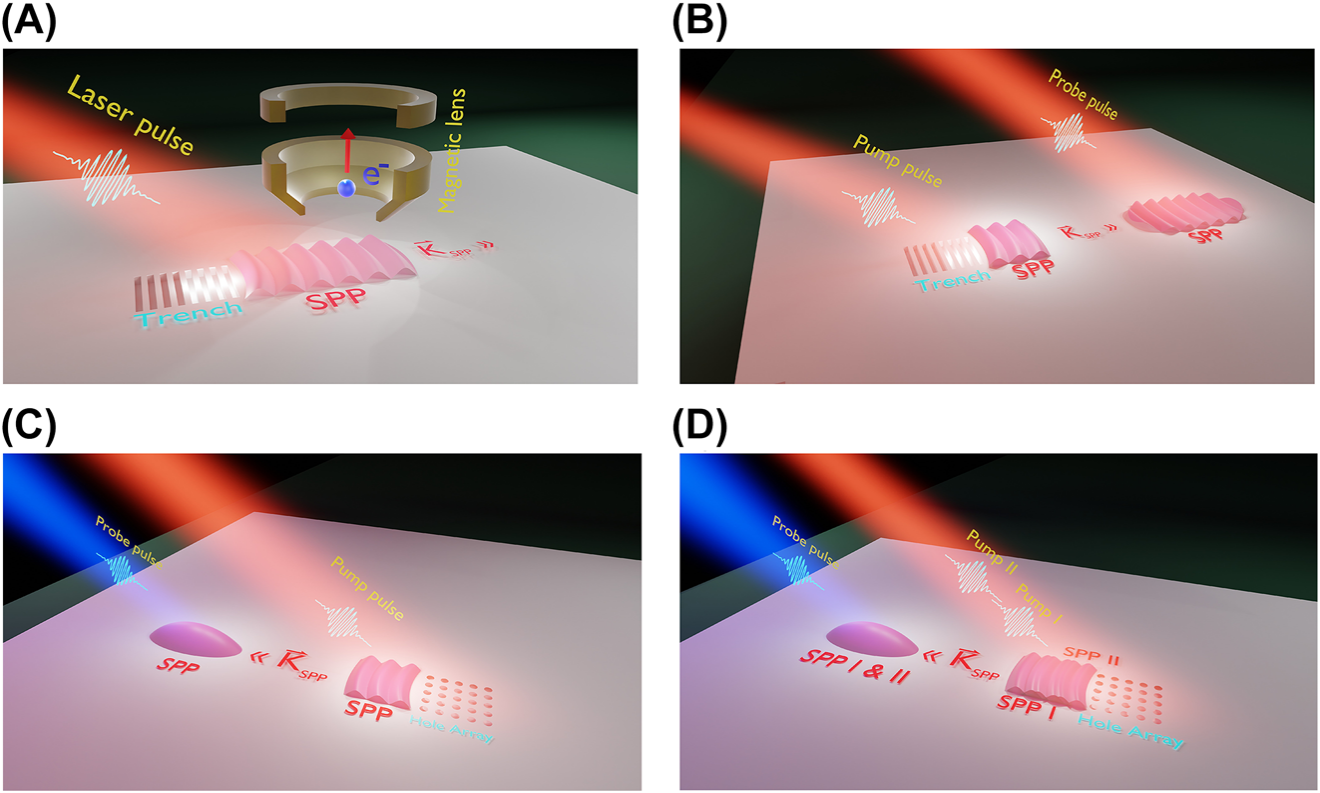
Photoemission electron microscopy (PEEM) modalities for surface plasmon imaging and manipulation: (A) a single femtosecond (fs) laser pulse impinging on a silver surface may couple to propagating surface-plasmon polariton (SPP) modes through surface imperfections or lithographically-etched structures. (B) Time-domain implementation of PEEM for SPP imaging. By introducing a time-delayed probe pulse that is spatially offset from the pump surface field, the SPP trajectory along the metal–air interface may be tracked in real time with fs resolution. (C) Reversing the SPP direction may be accomplished using periodic structures such as gratings or 2-dimensional periodic nano-hole arrays (2PGs) as depicted in (C). Multiple, phase-locked, counterpropagating SPPs may be prepared by introducing a second 800 nm pump field as depicted in (D). In (C and D), the use of a spatially offset 400 nm probe pulse significantly enhances the photoemission.

The photon-initiated SPP travels across the surface with a group velocity near, but not equal to, the speed of light in vacuum. As the SPP travels, it interacts with the surface field created by the initiating laser. This results in a beat pattern in the electric field often termed “self-interference” as depicted in Figure 1A. The typical photon source used is either a 400 nm or 800 nm femtosecond (fs) pulse derived from a mode-locked Ti:Sapphire laser. As the work functions of the metals that sustain SPPs are greater than the single photon energy at these wavelengths, the process of photoemission is necessarily nonlinear in nature and enhanced in regions where there are plasmonic excitations. Combined with the use of short fs pulses, this naturally leads to time-resolved implementations of this experiment that can monitor SPP trajectories stroboscopically by delaying a second fs probe pulse which interacts with the surface field to eject a photoelectron (Figure 1B–D). By imaging these photoelectrons, a map of the surface fields is registered as a time-resolved PEEM movie. Note that each frame includes contributions from the SPP, pump, and probe fields and their cross terms. Figure 1B–D displays a schematic of the different types of time-resolved experiments we employ to visualize and manipulate SPPs. The most straightforward implementation of a time-resolved experiment involves two fs pulses of the same color, traditionally either 800 nm or its second harmonic 400 nm (Figure 1B).

In most cases, the pump and probe pulses are interferometrically locked resulting in interferometric time-resolved PEEM (ITR-PEEM) as depicted in Figure 1B. Kubo et al. performed early ITR-PEEM measurements using phase-locked pairs of 400 nm fs pulses to observe SPP dynamics following excitation of trenches etched into silver-coated substrates [29]. Monitoring the SPP wave packet allows the determination of properties such as the SPP group velocity, lifetime, and dispersive broadening.

Similar experiments using 800 nm fs pulses impinging on a gold trench were performed by Gong et al. with one important difference [42]. In this report, the probe pulse was spatially offset to allow visualization of the SPP after it propagated some distance outside the region dominated by pump-SPP overlap. At 800 nm, the imaginary part of gold’s dielectric is small compared to the near-UV where real transitions dominate. As such, propagation may be visualized for significantly longer distances as the metal absorption channel which dominates SPP decay is minimized. Information on SPP velocities and lifetimes may be obtained through a straightforward analysis. One drawback of using 800 nm excitation in a multi-photon PEEM experiment is that a minimum of three and usually more photons must interact simultaneously to bridge the work function. Compared to 400 nm excitation, this results in much lower signal levels and adds some level of complexity to the signal analysis. One way to improve the signal level is to use a probe photon of a different color following excitation at 800 nm. Joly et al. provide an example of this technique which involves visualizing SPP propagation following initiation at a trench etched in a silver film [43]. Signal levels increased by more than an order of magnitude which allowed for the measurement of the 400 nm-initiated SPP, even with the strong damping mentioned above. This is mostly a result of the decreased nonlinear order of the photoemission because of the higher photon energy of the probe pulse. While the above experiment increases the signal levels dramatically, there are two main differences with single color two-pulse measurements. First, there are overlapping contributions from the 800 nm pulse, 400 nm pulse, 800 nm-initiated SPP, and 400 nm-initiated SPP which makes the analysis more cumbersome. Second, interferometric control is no longer an option as the mixing of a wave with its harmonic results in the detection of only a Gaussian envelope with width determined by the 400/800 nm pulse cross-correlation. Phase information is lost as a result.

The SPP dispersion approaches the light line at visible frequencies; therefore the momentum required to couple in the forward-scattered direction is trivial and may be provided by virtually any surface imperfection, asperity, or intentional structure.

**Figure 2:**
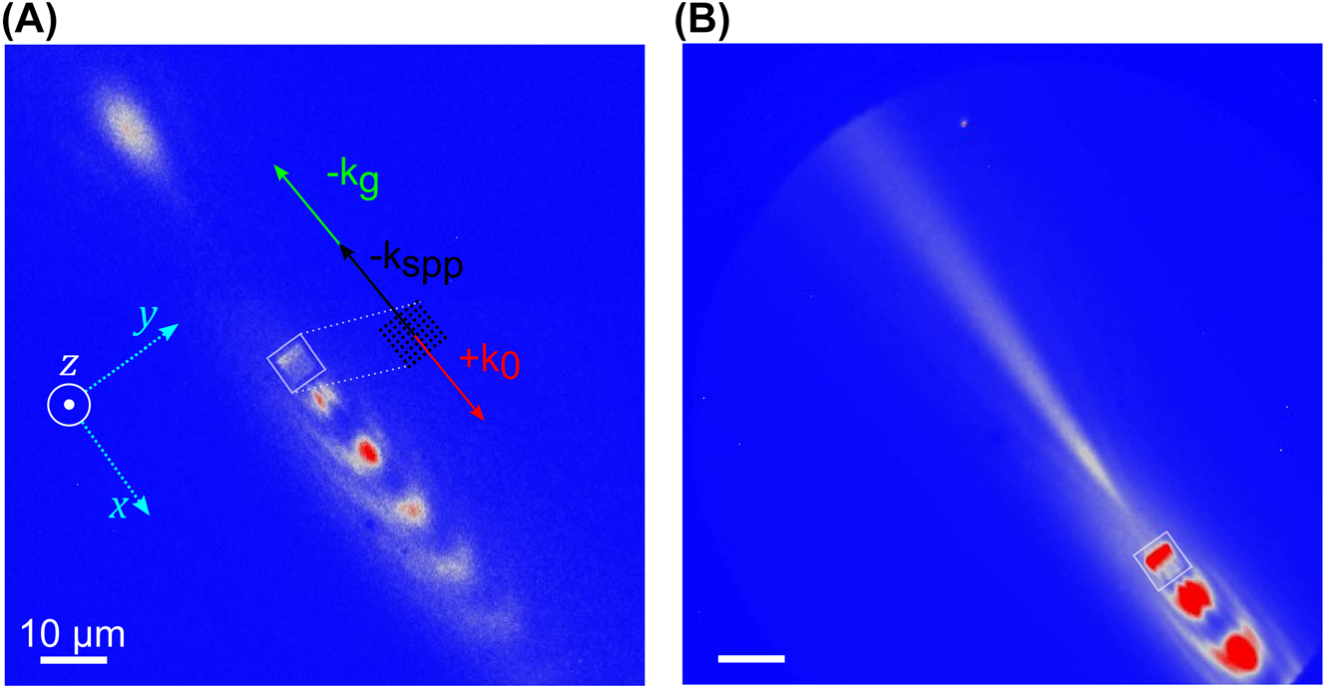
Simultaneous excitation of co- and counter-propagating SPPs using a 2-dimensional periodic nano-hole array (2PG) prepared through focused-ion beam (FIB) milling on a silver slab. The PEEM image shown in (A) is a single-frame extracted from a time-resolved movie tracking the SPP’s propagation. The beating pattern observed in the +*x* direction disappears in the −*x* direction due to temporal walk-off between the SPP and pump pulse. (B) By integrating the full trajectory, the asymptotic behavior of the SPP’s spatial profile becomes clear. It diverges after reaching a minimum spot size ∼5 µm from the array. Adapted with permission from ref. [44]. Copyright © 2019 American Chemical Society.

However, it is advantageous to be able to launch the SPP in arbitrary directions. This also has the effect of removing self-interference with the pump beam that complicates analysis and obscures the SPP field in the forward propagation direction. Crampton et al. employed two-dimensional plasmonic gratings (2PGs) to propel the SPP in the counter-propagating direction by utilizing the grating periodicity [44]. Consider an array with pitch of 400 nm and hole diameter of near 100 nm. The periodic structure provides a momentum of 2π/400 (nm^−1^) that can be either added to or subtracted from the in-plane momentum of the pump photon (sinθ*2π/800 (nm^−1^)). The use of 400 nm spacing in the grating recognizes that double the pump photon momentum is required to change the sign of the SPP direction from plus to minus, resulting in counter-propagation. A PEEM image demonstrating this effect is shown in Figure 2. Here, an 800 nm, 15 fs pulse is used to excite SPPs which are then detected using a 400 nm probe pulse that has been expanded to nearly cover the full field of view. By providing a time delay between the pump and probe pulses, the counter-launched SPP may be observed some distance away from the 2PG. The familiar self-interference pattern due to pump-SPP interactions is also apparent in the forward direction.

The use of periodic arrays such as that depicted in Figure 2 has multiple advantages when trying to control SPPs. First, arrays can lead to diffractive focusing effects, allowing for the tailoring of the spatial profile of the SPP field. Second, periodic structures supply an additional source of momentum and can be used to change the direction of the launched SPP. This can have several advantages. First, because the in-plane projection of the pump photon is traveling in the positive direction across the surface while the counter-propagating SPP is traveling in the negative direction, there is little interference between the two fields in either space or time. This provides a clearer way to visualize SPP propagation. Second, the counter-propagating SPP can now be monitored with a time-delayed probe beam to visualize and record the SPP spatial position and shape as a function of delay time. This permits the determination of the SPP group velocity, phase delay (if the probe is of the same color), and decay length. Thus, a measure of the real and imaginary dielectric function at the silver surface can be easily ascertained and the spatial profile can be visualized as the SPP propagates across the surface. It is evident in Figure 2A that the SPP spatial profile evolves as it propagates along the silver surface. Individual slices from the full trajectory show the spatial profile of the SPP as a function of time, essentially tracking the field envelope of the SPP. The profiles arise due to diffractive focusing where the array may be considered an apertured source. This provides additional degrees of freedom for manipulating the SPP beam parameters allowing the spatial profile to be controlled as well.

**Figure 3:**
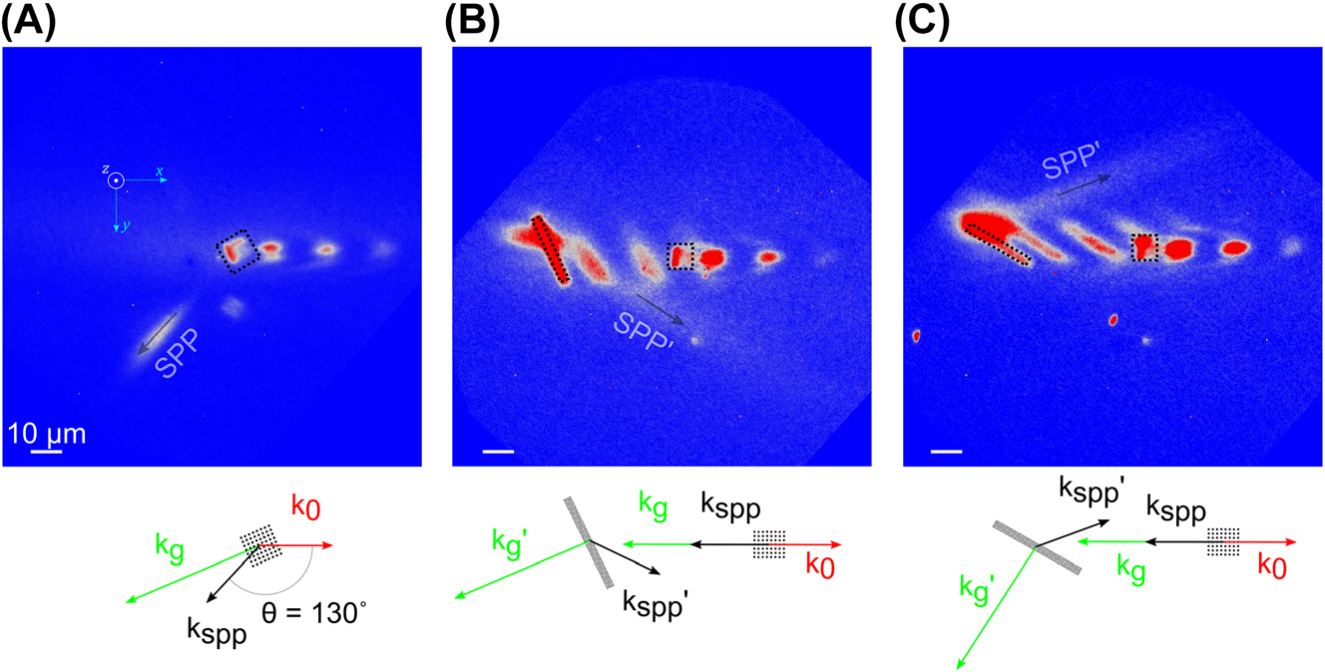
2PG-enabled SPP steering and manipulation. (A) The addition of transverse momentum allows counter-propagating SPPs to be launched in arbitrary directions in the negative half-space, as determined through vector phase-matching. The black square outlines the 400 nm pitch 2PG position used for coupling. (B and C) Counter-propagating SPPs launched along the −*x* unit vector using square 400 nm pitch 2PGs (black squares) interact with 565 nm pitch 2PG rectangular “mirrors” oriented at −30 and −60° (black rectangle) resulting in positive and negative refractory SPPs, respectively. Adapted from ref. [45] with the permission of AIP Publishing.

Another advantage of using 2PG structures for SPP coupling is that they can be used to launch photon-initiated SPPs in different directions. Figure 3A displays a case where the 450 nm pitch grating is situated at a 37° angle relative to the photon in-plane wave vector, resulting in a counter-propagating SPP at ∼130° from the in-plane wave vector [45]. Thus, it is possible to guide the nascent SPP in directions far different from the initiating photon in-plane wave vector by simply rotating the grating at an angle, therefore introducing a transverse component into the grating wave vector. In addition to guiding the SPP in a specific direction, 2PGs can be used as Bragg mirrors by taking advantage of their periodic structure. Figure 3B and C displays the results when a counter-propagating SPP is launched at a 2PG situated at differing angles. A 565 nm pitch 2PG when tuned to a 60-degree angle of incidence steers the SPP to 27 degrees off-normal while the 2PG tuned to 30° results in an SPP wave at −27°. These waves correspond to positive and negative refractory waves. Note, interference between the driving field and forward-launched SPPs can be observed beyond the excitation 2PG and in between the excitation and steering 2PG. The refractory waves result from the curvature of the 2PG band structure and provide a valuable way to control the SPP direction. For a 565 nm pitch grating at 45°, the SPP energy is partitioned into reflection and refraction channels at plus and minus 45°, respectively. A weak, but measurable amount of back-reflection is observed as well, constituting three-way SPP beam splitting (data not shown).

**Figure 4:**
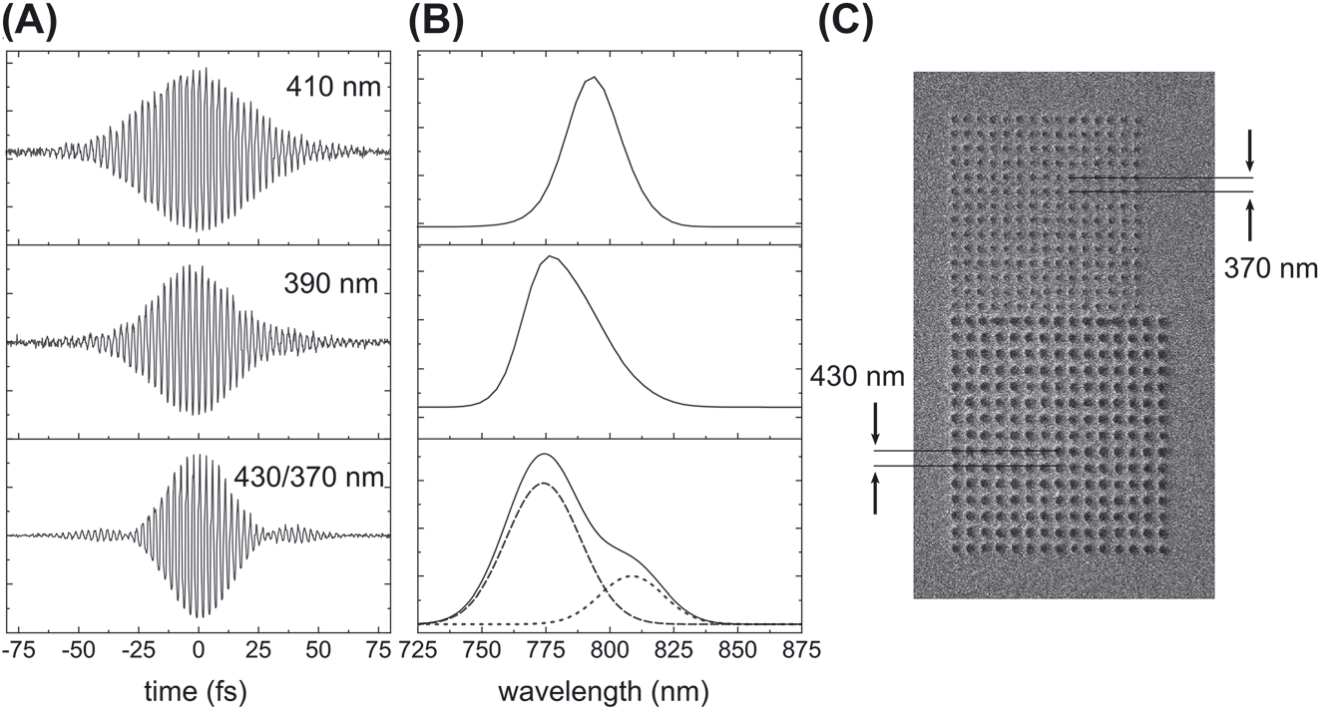
SPP spectroscopy and amplitude modulation. (A) Interferograms generated by autocorrelating counter-propagating SPPs launched through single- (410 (top), 390 nm (middle)) and a dual-pitch (430/370 nm (bottom)) 2PG displayed in (C). Tuning the 2PG pitch has the effect of modifying the frequency components that couple to the propagating SPP mode. (B) SPP spectra are recovered through Fourier transforms of the interferograms. For a dual-pitch coupler composed of adjacent 2PGs of different pitches, the PEEM signal becomes amplitude modulated (bottom panel of A). The discrete resonance intrinsic to the 430 nm 2PG appears as a side lobe on the main 370 nm state at 775 nm. Adapted from refs. [46], [48] (2021 and 2022, respectively); licensed under Creative Commons Attribution (CC BY) licenses.

The PEEM experiments described thus far demonstrate the utility of 2PGs for SPP generation and directional manipulation. Particularly, two-color pump-probe measurements provide a straightforward way to visualize SPP surface waves and record their trajectories. However, there is a cost relative to traditional single-color two-pulse ITR-PEEM because the two-color method does not preserve phase information. This limits the applicability of this approach for encoding and decoding information into SPPs from the excitation light field(s). Extending the two-color experiment to a three-pulse scheme as outlined in Figure 1D overcomes this limitation. In this scenario, two 800 nm phase-locked femtosecond pulses with variable delay are used to prepare SPP pairs while the third 400 nm pulse is used for increasing the surface field photoemission yield. Since the excitation of SPPs is a linear, coherent process, the phase relationship between the SPP and light field is trivial. This approach has the benefit of enhanced signal levels while also retaining phase information. In recent work, SPP autocorrelation measurements were carried out using the three-pulse, two-color ITR-PEEM approach [46]. Here, a pair of phase-locked 800 nm fs pulses was used to produce two counterpropagating SPPs via pitch-tuned 2PGs in silver. A magnified 400 nm pulse served to enhance the surface field photoemission. As mentioned above, the use of 400 nm light as a probe (for silver substrates) reduces the order of the photoemission from 3rd order to 2nd order. The resulting PEEM images are dominated by 2-photon photoemission. By controlling the interferometric delay between the pulses, the two SPPs are also phase-delayed as they propagate in the counter-propagating direction. The third blue pulse is delayed such that the electron emission corresponds to the SPP field at the spatial overlap point of the two phase-delayed SPPs. Thus, we acquire a time trajectory that sweeps the second SPP field through the “stationary” first SPP field at the overlap point. Figure 4 displays the results of these autocorrelations for 2PGs with pitches of 390 and 410 nm and their corresponding spectra. It is clear from Figure 4 that both the pulse structures and therefore frequency spectra can be controlled using appropriately selected 2PGs. More recent work using ITR-PEEM demonstrated how SPP can be steered in the forward-propagating direction using a trench coupler [47].

The coupling of light to 2PGs to form counter-propagating SPPs may be described through temporal convolution of the grating response function and the driving light field. As such, the frequency domain product of the laser spectrum and the 2PG resonance(s) yields the SPP spectrum, which may be recovered directly from Fourier transforms of the measured interferograms (Figure 4). If the 2PG resonances in the vicinity of the pump wavelength are different than the input pulse spectrum, the out-going SPP will be frequency filtered, allowing for SPP pulse shaping. Careful choice of 2PG then allows for frequency-modified (encoded) SPP pulses. Figure 4C demonstrates another way to use 2PGs as pulse shapers by combining two different pitch 2PGs into a super-array. The super-array is made by combining a 430 nm pitch array with a 370 nm pitch array in a lateral configuration [48]. Illumination with a femtosecond pulse results in two slightly laterally displaced SPPs. Using the autocorrelation technique above, the resulting field due to the interference of the two SPPs is shown in Figure 4A (bottom). The result is a modulated signal with sidelobes that appear near 30 fs from the main peak. The effect is analogous to signal transduction through amplitude modulation.

## 3 Conclusions

The SPP imaging and interrogation modalities we describe provide routes toward directional control, spatial structuring, and interferometric manipulation of plasmonic fields. These modalities represent a basis for more general ways to transfer information from coherent light fields to plasmonic waves through vectorial coupling. In the context of integrated photonic devices, utilizing the fundamental SPP manipulation tools we describe in conjunction with the plasmonic circuit elements that have been developed, in parallel, is a promising outlook [49]. To this end, an extension of the work we highlighted here would be to replace the lithographically prepared coupling structures altogether and, instead, rely on structured light and otherwise planar surfaces for SPP excitation, as has been demonstrated though four-wave mixing [50], [51]. Perhaps through pulse shaping technology, such virtual SPP couplers would enable programmatic amplitude and phase manipulation and expanded flexibility in nanoplasmonic circuits. New directions for structuring plasmonic fields using the intrinsic properties of materials have been shown in recent reports by Dai et al. and Ghosh et al. [52]–[54]. Through several reports, these works detail how topological field engineered and spin textured SPPs may be generated by taking advantage of spin–orbit interactions. These advancements bring further into focus the concepts of compound and multi-component devices that mimic existing all-electrical platforms in operation and design, but instead use plasmonic excitations for signal transportation.

On a final note, the commonly quoted description of plasmons as arising due to the collective motion of metal free electrons recognizes the classical nature of ensembles of oscillators. Pres et al. recently point out that, consequently, the majority of plasmonics research involves the description of plasmons as classical waves [55]. Their measurement of the discrete plasmon–polariton quantum states of a nanoslit resonator, enabled through 2D-nanoscopy in a PEEM, is a significant step forward for the development of quantum-enabled nanophotonics devices. The properties of quantum light that have opened up applications in encryption and single-photon manipulation further motivate future work in non-classical plasmonics [56]. We conclude by emphasizing that continued progress in the field of nanophotonics hinges on progress in each of these research directions and through their synergistic integration.
